# Supplementary material for: Achieving efficient power generation by designing bioinspired and multi-layered interfacial evaporator
Source: Nat Commun. 2022 Aug 29;13:5077. doi: 10.1038/s41467-022-32820-0 (PMC9424234; doi:10.1038/s41467-022-32820-0)
Supplement: Supplementary file 1 — Supplementary Information [file 41467_2022_32820_MOESM1_ESM.pdf]

Supplementary Materials for

Achieving efficient power generation by designing bioinspired and multi-layered interfacial evaporator

Zhuangzhi Sun[1,2,], Chuanlong Han[1], Shouwei Gao[3], Zhaoxin Li[1,*], Mingxing Jing[1], HaipengYu[2,*], Zuankai Wang[3,*]*

[1] *Province Key Laboratory of Forestry Intelligent Equipment Engineering, College of Mechanical and Electrical Engineering, Northeast Forestry University, Harbin 150000, People's Republic of China.*

[2] *Key Laboratory of Bio-based Material Science & Technology, Ministry of Education, Northeast Forestry University, Harbin 150000, People's Republic of China.*

[3] *Department of Mechanical and Biomedical Engineering, City University of Hong Kong, Hong Kong, People's Republic of China.*

E-mail: sunzhuangzhi@nefu.edu.cn; 2014211213@nefu.edu.cn; yuhaipeng20000@aliyun.com; zuanwang@cityu.edu.hk

Brief description of what this file includes:

Supplementary Figure 1. Bionic prototype and microstructures of moth-eye.

Supplementary Figure 2. Water evaporation properties under different moth-eye structure.

Supplementary Figure 3. Characteristics analyze of the top layer of the IENG.

Supplementary Figure 4. Voltage per unit area and performance comparison of the IENG.

Supplementary Figure 5. Water evaporation properties of the IENG at a 1.0 kW m^{-2}.

Supplementary Figure 6. Optimal analysis of IENG with bionic moth-eye surface and $CsPbBr_3$ type perovskite.

Supplementary Figure 7. Optical transmission mechanism of the IENG and moisture storage capacity.

Supplementary Figure 8. Vaporization enthalpy of the IENG.

Supplementary Figure 9. Relationship of the power generation performance, light intensity and water evaporation rate.

Supplementary Figure 10. The power generation performance of the IENG without evaporation.

Supplementary Figure 11. EDS and SEM images of the IENG in different salinity environment.

Supplementary Figure 12. Power generation performance of the IENG under different salinity.

Supplementary Figure 13. Water purification capacity and power generation of the IENG.

Supplementary Figure 14. The water evaporation experiment of the IENGs outdoor.

Supplementary Figure 15. Self-powered working system circuits & collection device illustration.

Supplementary Figure 16. Experiments of the IENG with different tested electrode materials.

Supplementary Note 1. Water evaporation rate measurement.

Supplementary Note 2. Evaluation of the energy conversion efficiency (η).

Supplementary Note 3. Relationship of electron velocity v_1 and solution flow rate v_0.

Supplementary Note 4. Relationships of the electron velocity v_1 with the short-circuit current (I_{SC}) and the open-circuit voltage (V_{OC}).

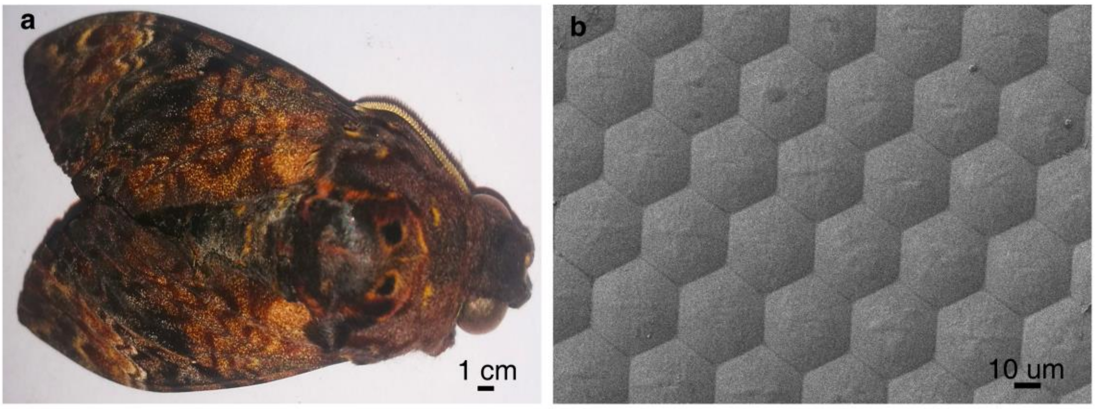

Supplementary Figure 1. Bionic prototype and microstructures of moth-eye. a Photograph of a bean hawk moth. **b** SEM image of the hexagonal structure inside the moth-eye.

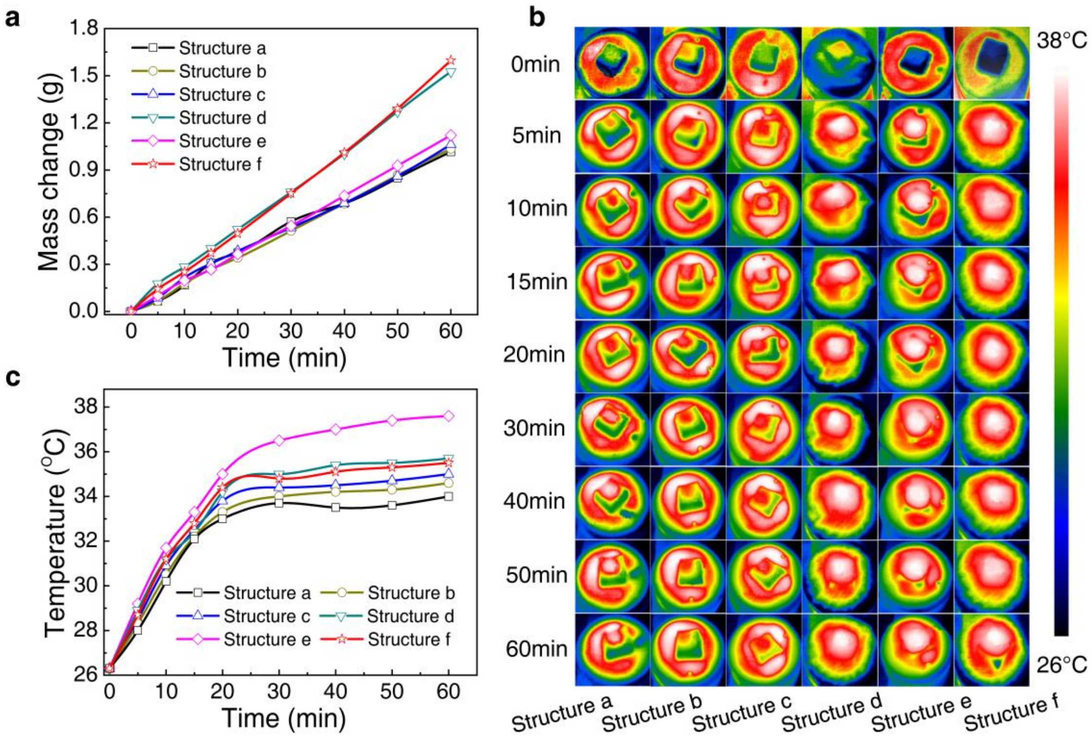

Supplementary Figure 2. Water evaporation properties under different moth-eye structure. **a** Evaporation of bionic moth-eye with different shapes. **b** Temperature changes on the surface of bionic moth-eye with different shapes recorded by infrared camera under 1.0 kW m^{-2} light intensity. **c** Temperature on the surface of bionic moth-eye with different shape.

Structure a: bionic moth-eye structure with low concave distribution density. **Structure b:** bionic moth-eye with concave distribution and medium density. **Structure c:** bionic moth-eye with high concave distribution density. **Structure d:** bionic moth-eye with a convex distribution density and a small size shape. **Structure e:** bionic moth-eye with convex distribution density and large size shape. **Structure f:** the concave-convex shape bionic moth-eye with a high distribution density and a small size shape.

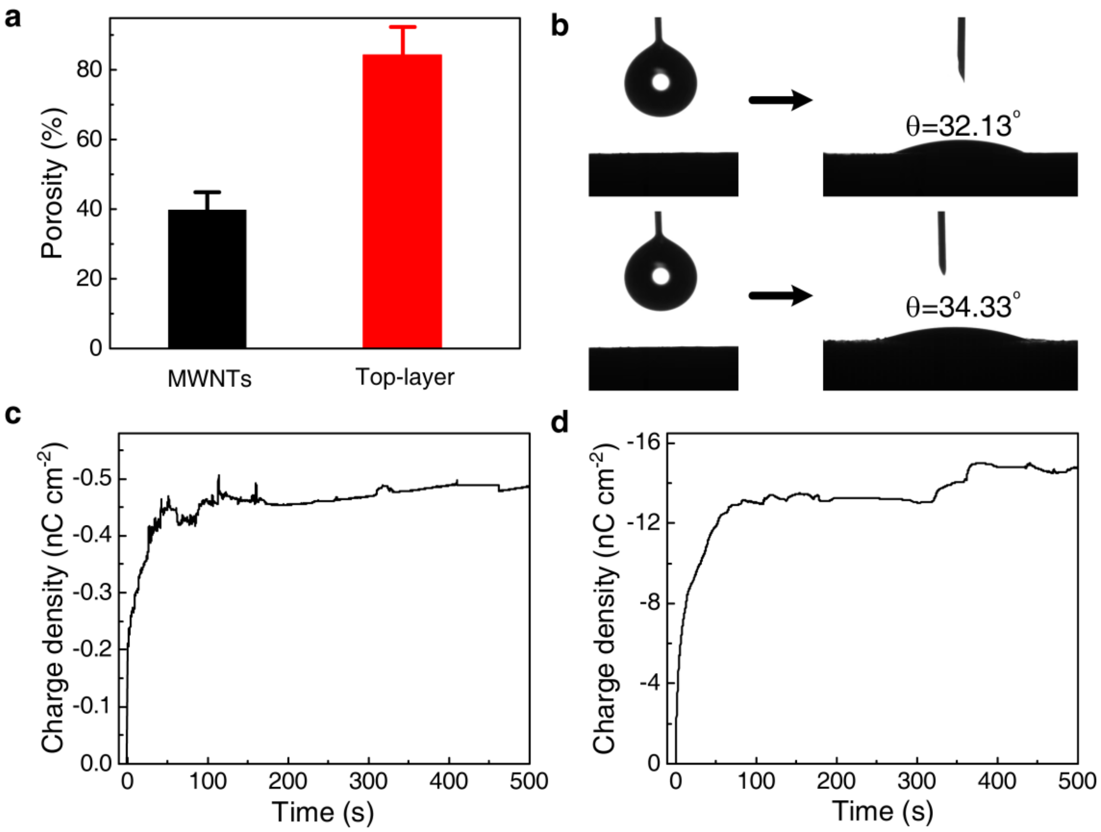

Supplementary Figure 3. Characteristics analyze of the top layer of the IENG. a The porosity of the top layer. **b** Hydrophilic properties of the top layer and the middle layer. **c** Surface charge distribution of the dried top layer. **d** Surface charge distribution of the wet top layer. The error bars represent standard deviations.

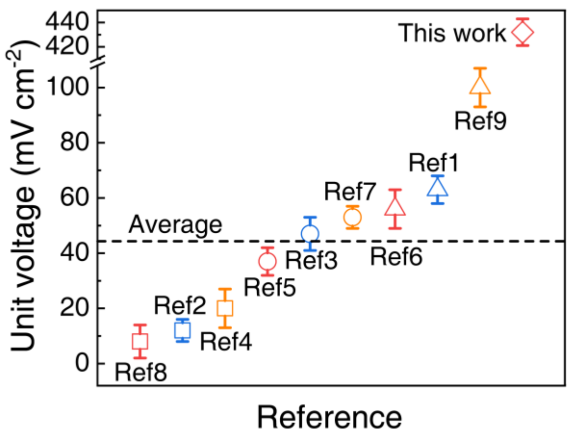

Supplementary Figure 4. Voltage per unit area and performance comparison of the IENG.

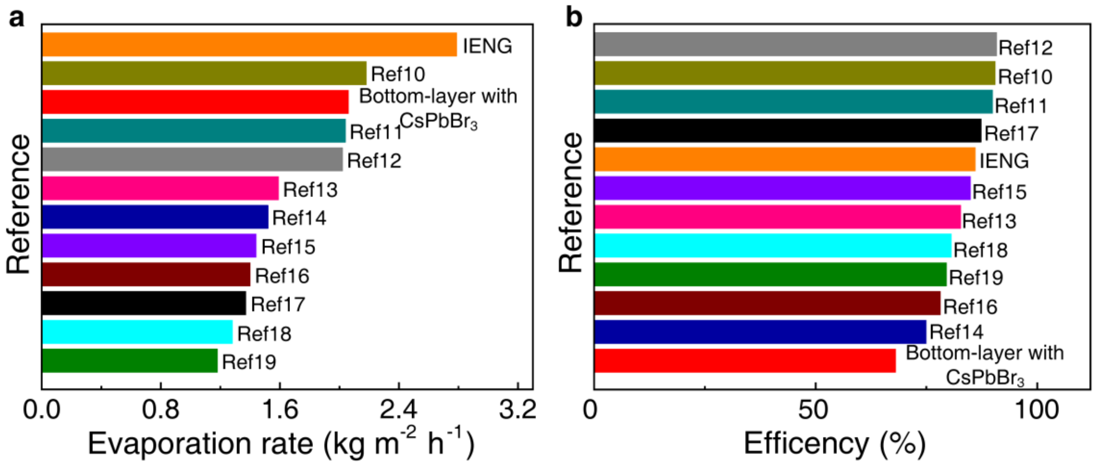

Supplementary Figure 5. Water evaporation properties of the IENG at a 1.0 kW m^{-2}. a The water evaporation rate of the IENG, the bottom layer with $CsPbBr_3$ and other solar-driven evaporators. **b** Comparison of energy conversion rates between the IENG, the bottom layer with $CsPbBr_3$ and other evaporators.

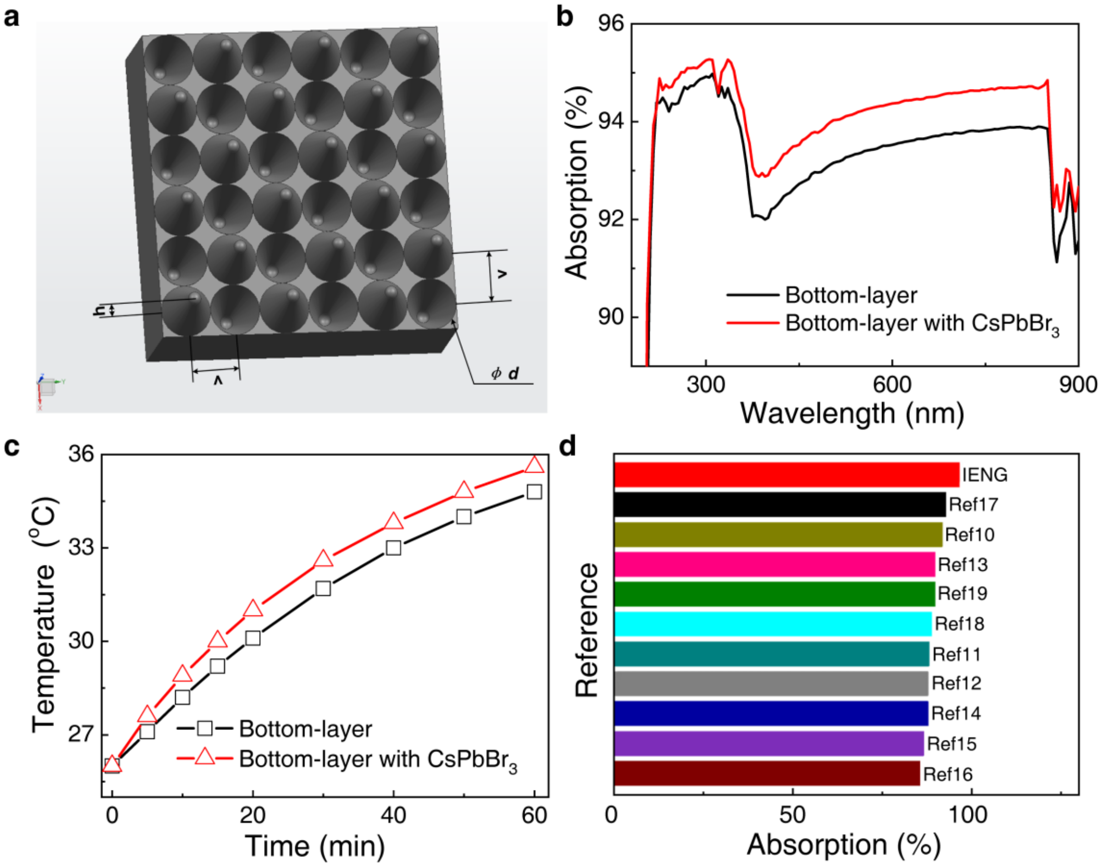

Supplementary Figure 6. Optimal analysis of the IENG with bionic moth-eye surface and $CsPbBr_3$ type perovskite. a Theoretical model of the moth-eye structure. *Λ* is the period of moth-eye structure. *h* is the depth of moth-eye structure. *d* is the bottom diameter of moth-eye structure. **b** Light absorption efficiency of the bottom layer with and without perovskite at the wavelength range of 190 nm-900 nm. **c** Surface temperature curves of the bottom layer with and without perovskite under a solar light intensity of 1.0 kW m^{-2}. **d** Comparison of light absorption efficiency between the IENG and other evaporators.

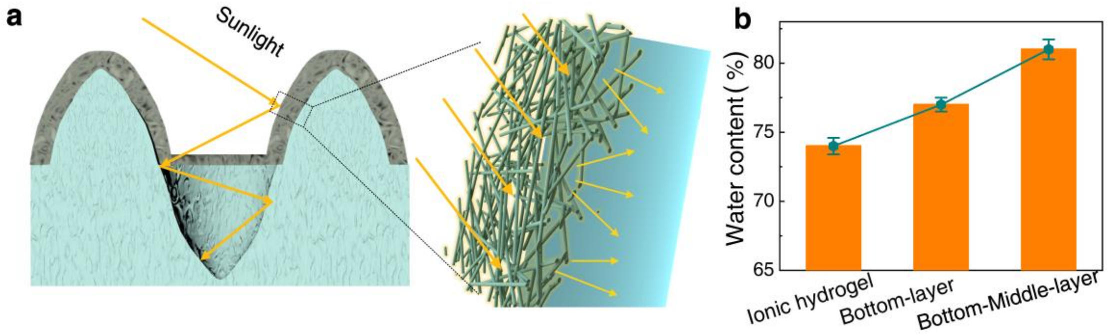

Supplementary Figure 7. Optical transmission mechanism of the IENG and moisture storage capacity. a Working principle of optical transmission with enhanced bionic moth eye structure. **b** Moisture storage capacity of the IH, the bottom layer, the bottom-middle-layer. The error bars represent standard deviations.

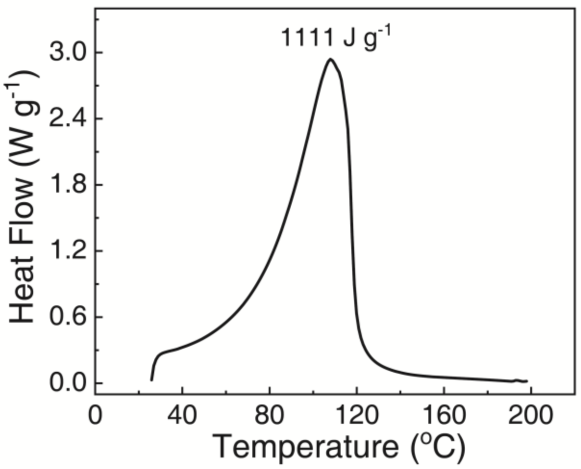

Supplementary Figure 8. Vaporization enthalpy of the IENG.

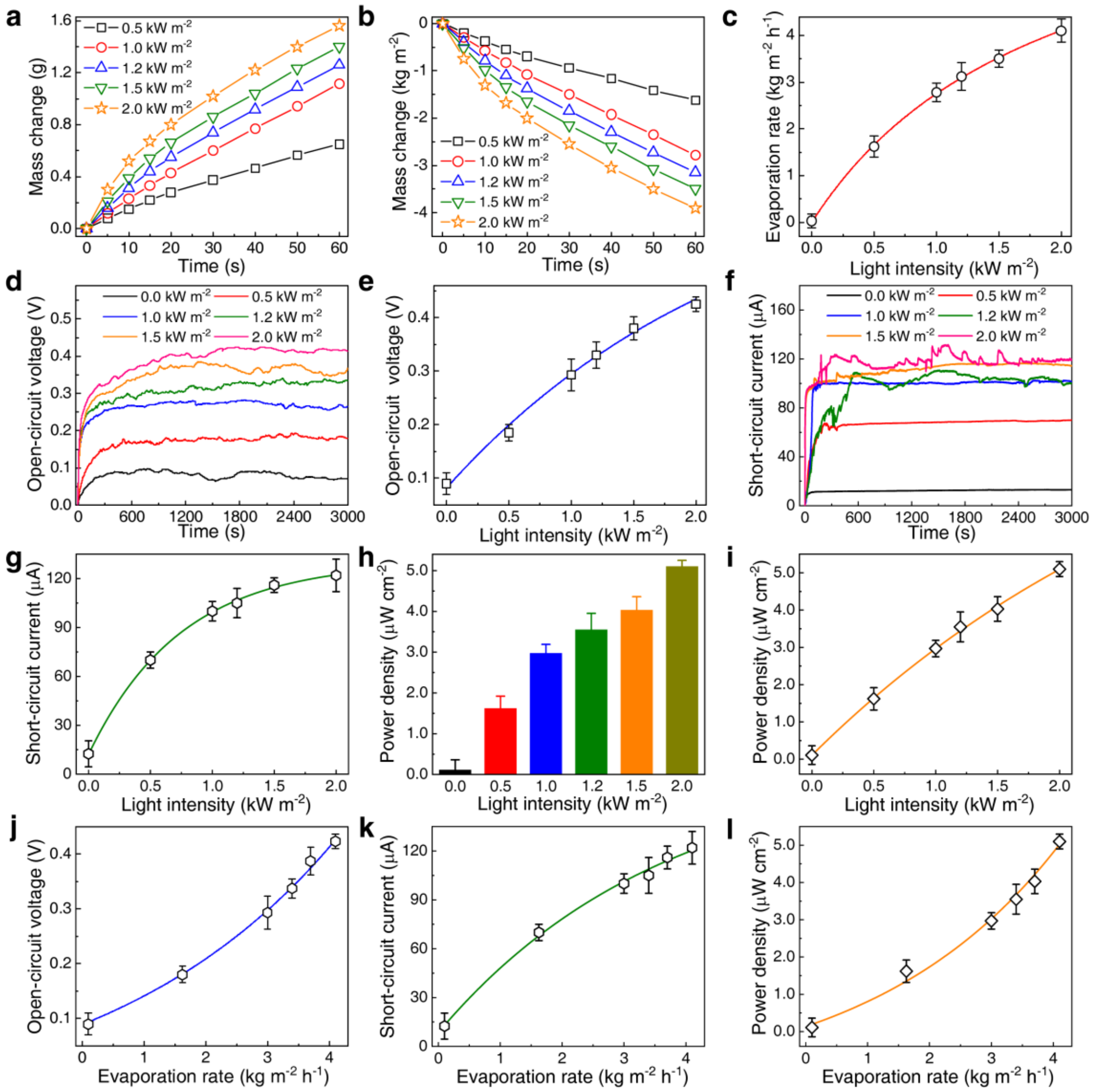

Supplementary Figure 9. Relationship of the power generation performance, light intensity and water evaporation rate. a Mass loss of the IENG under different light intensities. **b** Evaporation rate of the IENG under different light intensities. **c** Relationship between the evaporation rate and the light intensity. **d** Open-circuit voltage under different light intensities **e** Relationship between the open-circuit voltage and the light intensity. **f** Short-circuit-current under different light intensities. **g** Relationship between the short-circuit current and the light intensity **h** Power density under different light intensities. **i** Relationship between the power density and the light intensity. **j-l** Relationship between power generation and water evaporation rate under different light intensities. The error bars represent standard deviations.

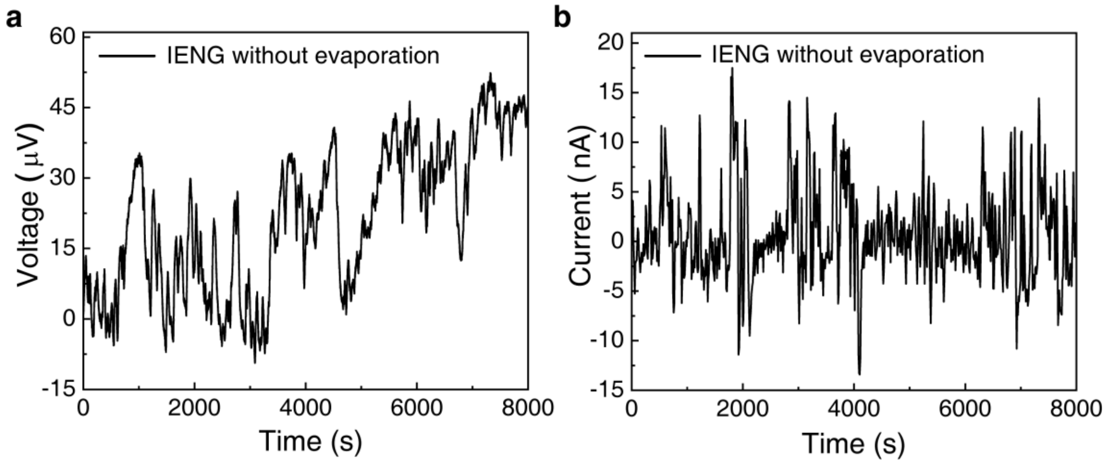

Supplementary Figure 10. The power generation performance of the IENG without evaporation. a The open-circuit voltage of the IENG without evaporation. **b** The short-circuit current of the IENG without evaporation.

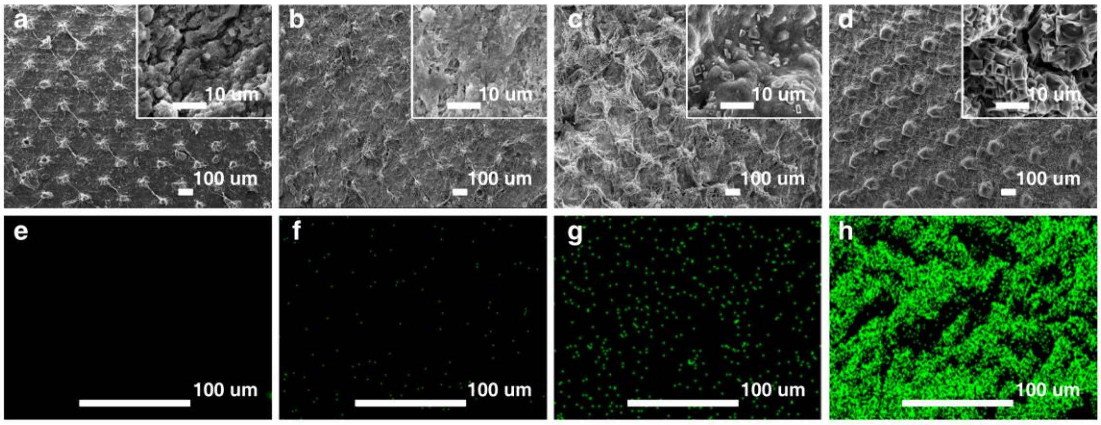

Supplementary Figure 11. EDS and SEM images of the IENG in different salinity environment. a, e Contrast sample. **b, f** Samples soaking in sea water for 72h. **c, g** Samples evaporating for 72h. **d, h** Samples evaporating for more than 300h.

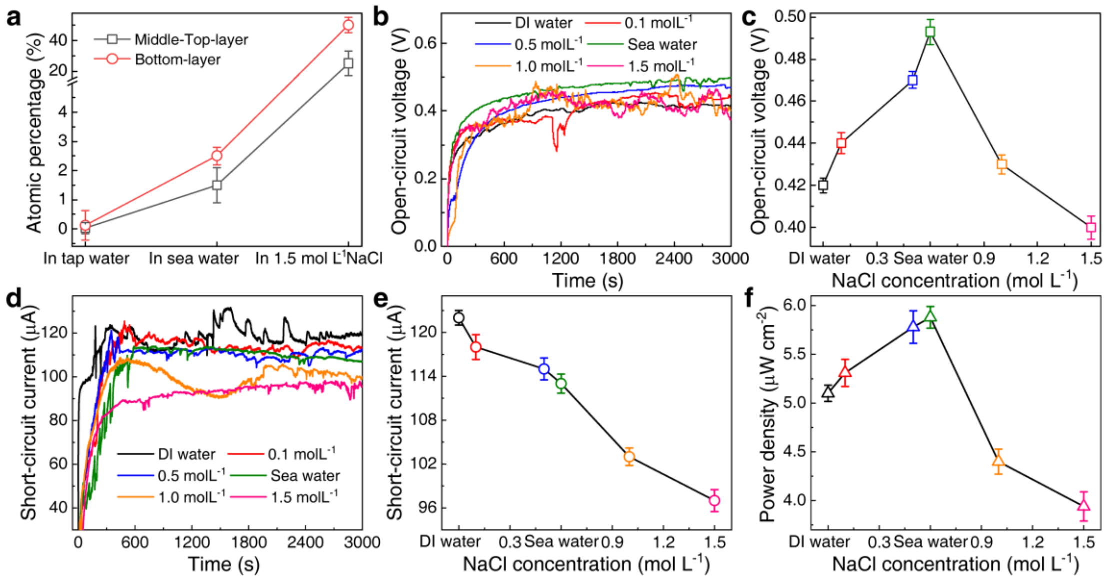

Supplementary Figure 12. Power generation performance of the IENG under different salinity. a Residual amount of Na after evaporation of the IENG under different salinity. **b** Open-circuit voltage generated by the IENG under different salinity. **c** Variation of open-circuit voltage produced by the IENG under different salinities. **d** Short-circuit current generated by the IENG under different salinity. **e** Variation of short-circuit current produced by the IENG under different salinity. **f** Power density produced by the IENG under different salinity. The error bars represent standard deviations.

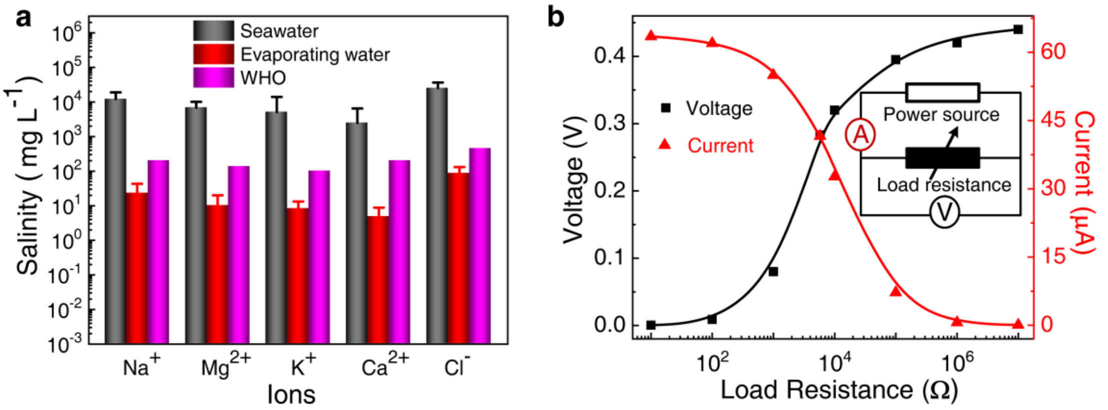

Supplementary Figure 13. Water purification capacity and power generation of the IENG. a Salinity before and after water purification. Sea water with an average salinity of ∼1 wt % is collected from the Bohai Sea, China. **b** Current and voltage generated via the IENG connected to a variable external load. The error bars represent standard deviations.

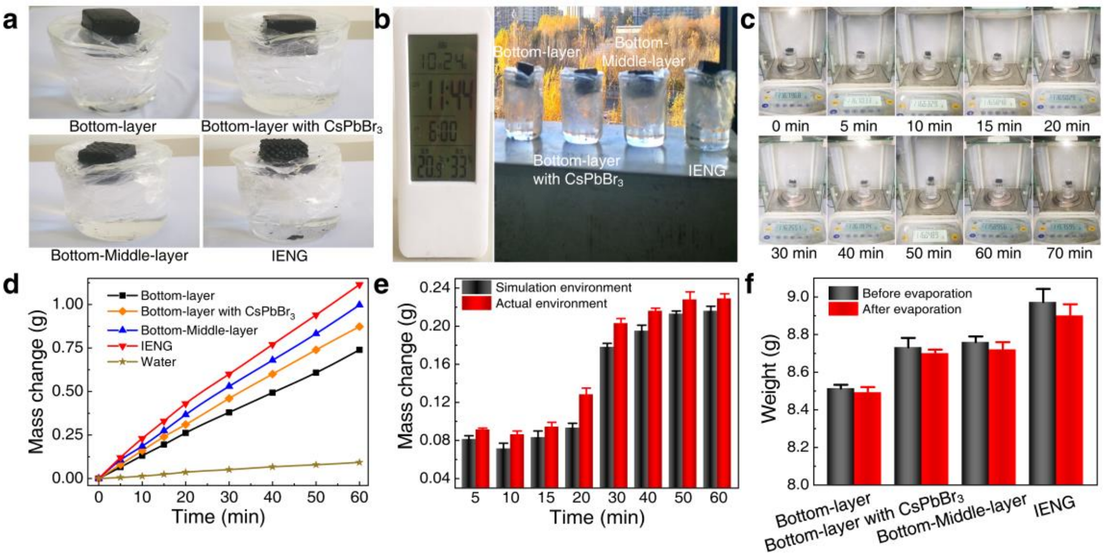

Supplementary Figure 14. The water evaporation experiment of the IENGs outdoor. a Water evaporation experiment of the IENG. **b** The water evaporation condition of the IENGs. **c** Mass loss of the IENGs under normal light condition. **d** The quality water loss of changes over time under the sunlight condition. **e** Mass loss of the IENG over time under simulated condition and actual sunlight. **f** Mass change of the IENGs before and after water evaporation. The error bars represent standard deviations.

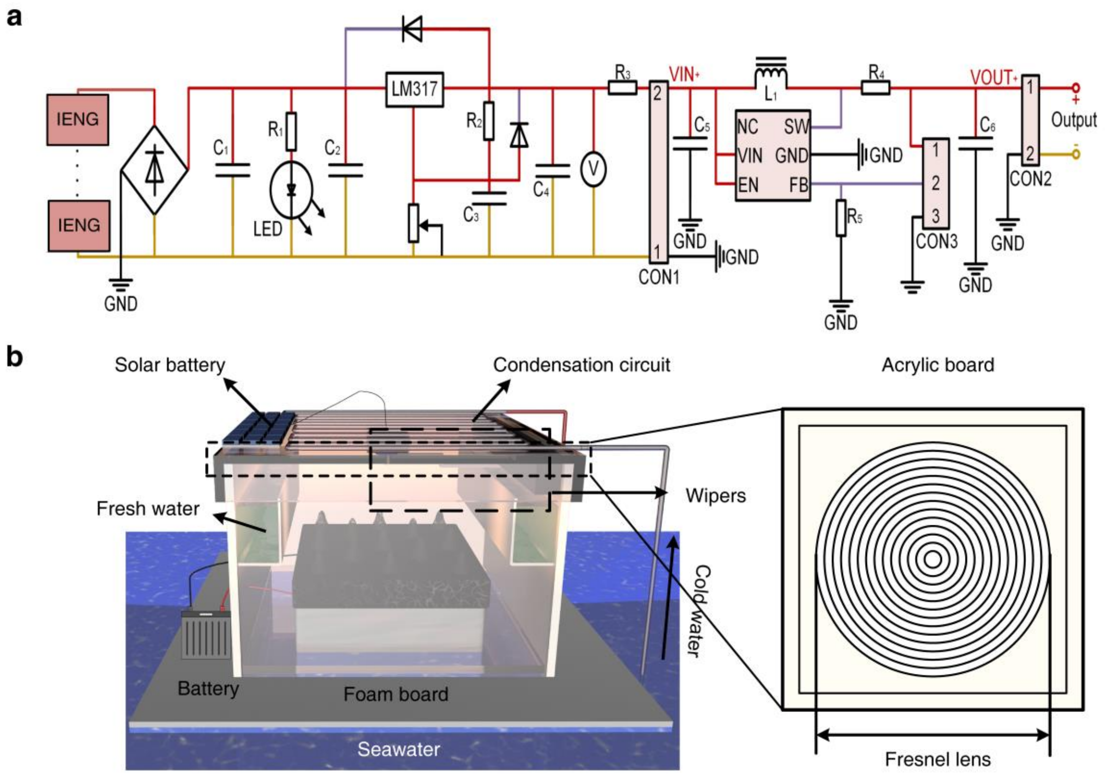

Supplementary Figure 15. Self-powered working system circuits and collection device illustration. a Schematic diagram of the self-powered working system circuit. **b** The collection device illustration for ocean power generation and fresh water production.

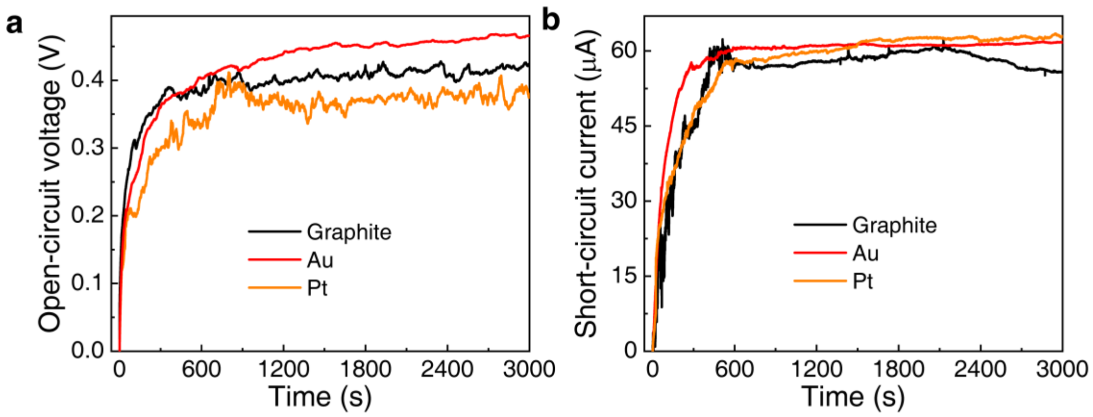

Supplementary Figure 16. Experiments of the IENG with different tested electrode materials. **a** Open-circuit voltage of the IENG with different tested electrode materials. **b** Short-circuit current of the IENG with different tested electrode materials.

Supplementary Note 1. Water evaporation rate measurement

The water evaporation rate (E.R.) is given by the following equation

$$\mathrm{E.R.} = 10000\frac{m}{st} \tag{S1}$$

where m is the mass reduction unit (kg), s is the upper surface area unit (cm^2), and t is the evaporation time (h).

Supplementary Note 2. Evaluation of the energy conversion efficiency (*η*)

$$\eta = \frac{\mathrm{E.R.} \times h_{\mathrm{lv}}}{C_{\mathrm{opt}} \times q_{\mathrm{i}}} \tag{S2}$$

where E.R. is the evaporation rate, h_{lv} denotes the total enthalpy of liquid-vapor phase change, C_{opt} stands for the optical concentration, and q_{i} is solar irradiation (1kW m^{-2}).

Supplementary Note 3. Relationship of electron velocity v_1 and solution flow rate v_0[20,21]

$$v_1 = \frac{\mu E k_B T}{2\sigma_C L} \ln \frac{v_0}{v_C} \tag{S3}$$

$$v_C = \frac{\beta k_B T}{\alpha \sigma_C a L} \exp\left(-\frac{a\sigma_C^2 L^2}{E k_B T} \right) \tag{S4}$$

where μ is electron migration rate. L, a and E are length, thickness and elastic modulus of the model slider, respectively. k_B is Boltzmann constant. v_0 is solution flow rate. v_1 is electron velocity. α is linear factor in the Newtonian fluid model ($v=\alpha v_0$). β is pre-exponential factor for calculating activation energy required for slippage (Arrhenius equation). σ_C is the critical elastic deformation tension.

Supplementary Note 4. Relationships of the electron velocity v_1 with short-circuit current (I_{SC}) and open-circuit voltage (V_{OC})[20,21]

$$I_{SC} = \frac{\kappa}{1+\kappa} 2\pi r e n_e v_1 \quad \text{(S5)}$$

$$V_{OC} = \kappa R_0 2\pi r e n_e v_1 \quad \text{(S6)}$$

where κ is constant related to the interaction between ions and carriers, r is radius of MWNTs, e is electronic charge, n_e is carrier concentration in MWNTs and R_0 is resistance of MWNTs.

Supplementary References

1. Ding, T. et al. All-printed porous carbon film for electricity generation from evaporation-driven water flow. *Adv. Funct. Mater.* **27**, 1700551 (2007).
2. Zhou, X. et al. Harvesting electricity from water evaporation through microchannels of natural wood. *ACS Appl. Mater. Inter.* **12**, 11232-11239 (2020).
3. Sun, J. et al. Electricity generation from a Ni-Al layered double hydroxide-based flexible generator driven by natural water evaporation. *Nano Energy* **57**, 269-278 (2019).
4. Hou, B. et al. Flexible graphene oxide/mixed cellulose ester films for electricity generation and solar desalination. *Appl. Therm. Eng.* **163**, 114322 (2019).
5. Xiao, P. et al. Exploring interface confined water flow and evaporation enables solar-thermal-electro integration towards clean water and electricity harvest via asymmetric functionalization strategy. *Nano Energy* **68**, 104385 (2020).
6. Li, L. et al. Sustainable and flexible hydrovoltaic power generator for wearable sensing electronics. *Nano Energy* **72**, 104663 (2020).
7. Zhang, G. et al. Harvesting environment energy from water-evaporation over free-standing graphene oxide sponges. *Carbon* **148**, 1-8 (2019).
8. Dao, V. D., Vu, V. H. & Choi, H. S. All day limnobium laevigatum inspired nanogenerator self-driven via water evaporation. *J. Power Sources* **448**, 227388 (2020).
9. Qin, Y. et al. Constant electricity generation in nanostructured silicon by evaporation-driven water flow. *Ange. Chem. Int. Edit.* **59**, 10619-10625 (2020).
10. Hu, G. et al. Salt-resistant carbon nanotubes/polyvinyl alcohol hybrid gels with tunable water transport for high-efficiency and long-term solar steam generation. *Energy Technol.* **8**, 1900721 (2020).
11. Long, Y. et al. Carrot-inspired solar thermal evaporator. *J. Mater. Chem. A* **7**, 26911-26916 (2020).
12. Liu, C. et al. Accelerated solar steam generation for efficient ions removal. *J. Colloid Inter. Sci.* **560**, 103-110 (2020).
13. Yu, F. et al. Quasimetallic molybdenum carbide-based flexible polyvinyl alcohol hydrogels for enhancing solar water evaporation. *Adv. Mate. Inter.* **6**, 1901168 (2019).
14. Guo, Z. et al. Stable metallic 1T phase engineering of molybdenum disulfide for enhanced solar vapor generation. *Sol. Energ. Mat. Sol. C.* **204,** 110227 (2020).
15. Zha, X. et al. Flexible anti-biofouling MXene/cellulose fibrous membrane for sustainable solar-driven water purification. *Acs Appl. Mater. Inter.* **11**, 36589-36597 (2019).
16. Yang, Y., Sui, Y., Ca, Z. & Xu, B. Low-cost and high-efficiency solar-driven vapor generation

using a 3D dyed cotton towel. *Glob. Chall.* **3**, 1900004 (2019).

17. Wang, H. et al. Enhanced photothermal conversion by hot-electron effect in ultrablack carbon aerogel for solar steam generation. *Acs Appl. Mater. Inter.* **11**, 42057-42065 (2019).
18. Li, Z. et al. Broadband-absorbing WO_3-x nanorod-decorated wood evaporator for highly efficient solar-driven interfacial steam generation. *Sol. Energ. Mater. Sol. C.* **205**, 110254 (2020).
19. Qi, Q. et al. High-efficiency solar evaporator prepared by one-step carbon nanotubes loading on cotton fabric toward water purification. *Sci. Total Environ.* **698**, 134136 (2020).
20. Liu, K. et al. Induced potential in porous carbon films through water vapor absorption. *Angew. Chem. Int. Edit.* **55**, 8003-8007 (2016).
21. Zhou, X. et al. Harvesting electricity from water evaporation through microchannels of natural wood. *ACS Appl. Mater. Inter*. **12**, 11232-11239 (2020).
